# Comprehensive Analysis of Gene Expression Profiles and DNA Methylome reveals *Oas1, Ppie, Polr2g* as Pathogenic Target Genes of Gestational Diabetes Mellitus

**DOI:** 10.1038/s41598-018-34292-z

**Published:** 2018-11-02

**Authors:** Yan Zhang, Tiancheng Zhang, Yunyan Chen

**Affiliations:** 1 Department of Obstetrics & Gynecology, Renji Hospital, School of Medicine, Shanghai Jiaotong University, Shanghai, P. R. China; 2 Key Lab of Reproduction Regulation of NPFPC-Shanghai Institute of Planned Parenthood Research (SIPPR), Fudan University Reproduction and Development Institution, Shanghai, China

## Abstract

Gestational Diabetes Mellitus (GDM) has a high incidence of pregnancy, which seriously affects the life quality of pregnant women and fetal health. DNA methylation is one of the most important epigenetic modification that can regulate the gene expression level, and thus affect the occurrence of various diseases. Increasing evidence has shown that gene expression changes caused by DNA methylation play an important role in metabolic diseases. Here we explored the mechanisms and biological processes that affect the occurrence and development of GDM through analyzing the gene expression profiles and DNA methylation data of GDM. We detected 24,577 differential CpG sites mapping to 9339 genes (DMGs, differential methylation gene) and 931 differential expressed genes (DEGs) between normal samples and GDM samples. GO (gene ontology) and KEGG (Kyoto Encyclopedia of Genes and Genomes) pathway analysis of 326 overlapping genes between DMGs and DEGs showed obvious enrichment in terms related to metabolic disorders and immune responses. We identified *Oas1, Ppie, Polr2g* as possible pathogenic target genes of GDM by combining protein-protein interaction analysis. Our study provides possible targets for early diagnosis of GDM and information for clinical prevention of abnormal fetal development and type 2 diabetes.

## Introduction

Gestational Diabetes Mellitus (GDM) is caused by the failure of pancreatic β cells to meet the increased insulin requirements and metabolic demands of pregnancy^1^. In addition to abnormal glucose tolerance and insulin resistance, GDM patients often show complications including chronic systemic inflammation^2^, increased leptin concentration^3^, and reduced adiponectin content^4^. High risks of Type2 diabetes among women with GDM history and abnormal fetus development caused by GDM emphasized the importance of deciphering the molecular mechanisms governing the progression of GDM. GWAS^5^ and metabonomic studies these years shed light on the genomic propensity and metabolic stimulus causing GDM, but the transcriptional biomarkers of peripheral organ and the epigenetic regulatory mechanism of GDM were less studied.

As an important epigenetic modification, DNA methylation represses transcription by inhibiting the binding of specific transcription factors as well as by recruiting methyl-CpG-binding repressive chromatin remodelers^6^. DNA methylation involves in various biological processes including cell proliferation, cell fate transition, X chromosome inactivation, genomic imprinting and chromosome stability^6^. Benefiting from the advances of methylation sequencing technology and big data storage and analytics approaches, more and more clues on the mammalian development and diseases progression have been dug out. Information provided by systematic methylome analysis combining other omic studies is a huge booster for the development of the Precision Medicine^7–10^.

The methyl donor for DNA methylation is the intermediate product of one-carbon metabolism, and increasing evidence have shown that DNA methylation can response to nutritional and environmental influences and regulate gene expression pattern accordingly^11^. For instance, DNA methylome changes were recognized as a faithful readout in tis-sues such as skeletal muscle and adipose in obese people with metabolic abnormalities^12,13^. Furthermore, genome-wide methylome and transcriptome analysis in hepatocyte isolated from normal people and patients with type 2 diabetes revealed that genes involved in hepatic glycolysis and insulin re-sistance were abnormally hypomethylated and thus overexpressed in liver^14^.Therefore, it is reasonable to speculate that the placenta, as a peripheral tissue susceptible to metabolic disorders, its DNA methylation regulation plays an important role in the pathology.

In this study, we confirmed genes related to immune response and metabolic pathways were differentially expressed between healthy gestational women and women with GDM. Differences in expression profile were regulated at least in a great part by DNA methylation level according to our comprehensive DNA methylome studies. We also integrated protein-protein interaction network analysis in our study and find Oas1, Ppie, Polr2 are important GDM target genes. Our study will shed light on early diagnosis of GDM and contribute to the prevention of abnormal neonatal development caused by GDM.

## Results

### Differential methylation sites

**Figure 1:**
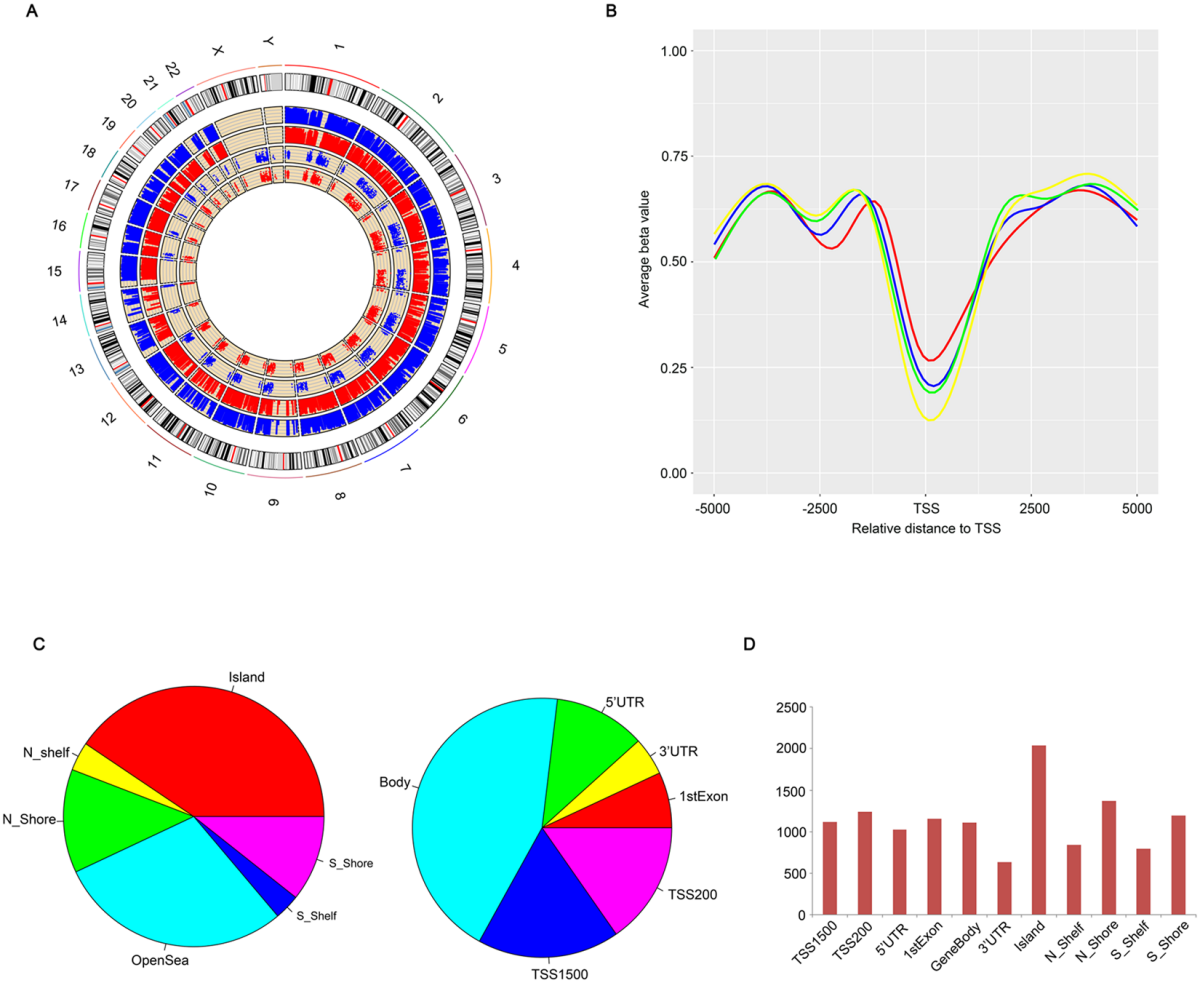
Overview of DNA methylation status across samples. (**A**) From the outside to the inside are the genome positions by chromosomes, whole methylation level of the control group (blue bars) and the GDM group (red bars), the overall expression level of the control group (blue dots) and the GDM group (red dots). (**B**) Distribution of DNA methylation across genes with different expression level along 5 kb upstream and downstream of the TSS (transcription start site). Red line: genes with lowest to lower quartile average expression value of all samples; blue line: genes with lower quartile to median average expression value of all samples; green line: genes with median to upper quartile average expression value of all samples; yellow line: genes with upper quartile to largest average expression value of all samples. (**C**) Distribution of differential methylated sites relative to CpG Island and multiple gene elements. (**D**) Distribution of DMG number obtained by region-level analysis.

Raw methylation data was preprocessed by filtering out high noise methylation sites and samples containing high noise regions. As a result, 473,281 out of 485,577 (97.47%) sites and all of the samples passed the quality control process, indicating high data quality. We firstly assessed differences in overall DNA methylation patterns between normal and DMG patients placental tissues as shown in Fig. 1A. Differential methylation analysis detected 24,577 differential CpG sites corresponding to 9339 genes (DMG, differential methylation gene) across samples. We then ordered all genes detected according to their expression level and divided them into four groups, namely 0–25%, 25–50%, 50–75%, 75–100%. As shown in Fig. 1B, methylation level negatively correlates to gene expression level which is in constant with their roles in repressing gene transcription. Besides, DNA methylation level decreased dramatically upstream the TSS, increased sharply toward the gene body regions, and remained at a low plateau until the 3′end of the gene body and showed the lowest point at TSS (Fig. 1B).

The function of DNA methylation relay on the local CpG content and position relative to genes^15^. To discover the regulatory effect of this DNA modification in our system, we mapped DMSs between two groups of samples to neighborhood location, including CpG islands(CGIs), their shores (±2 kb of CGIs) and shelves (±2–4 kb of shores), and to functional genomic regions, including distal promoters (±1.5 kb), proximal promoters (±0.2 kb), gene bodies, untranslated regions containing 3′ UTR (define regions) and 5’ UTR (define regions). The statistical results are detailed in the pie chart of Fig. 1C. Briefly, DMSs located most in CpG island regions in contrast to their upstream and downstream regions including the shores and shelves. When mapping into gene regions, gene body regions contained the most DMSs relative to their expression regulatory regions. We conducted region-level analysis to reveal the DMG distribution pattern across different genetic regions. Methylation levels in each gene element were compared among samples according to their beta value (see methods). Statistical result was shown in Fig. 1D. Most DMG screened out by methylation level changes in CpG island region enriched in promoters. Up-regulated DNA methylation on promoters of tumor suppressor genes is common in many kinds of malignant tumor. Taken together, our data imply that DNA methylation is highly differentially regulated and may function in regulating pathogenic gene expression in DMG patient placenta.

### Differential expressed genes

**Figure 2:**
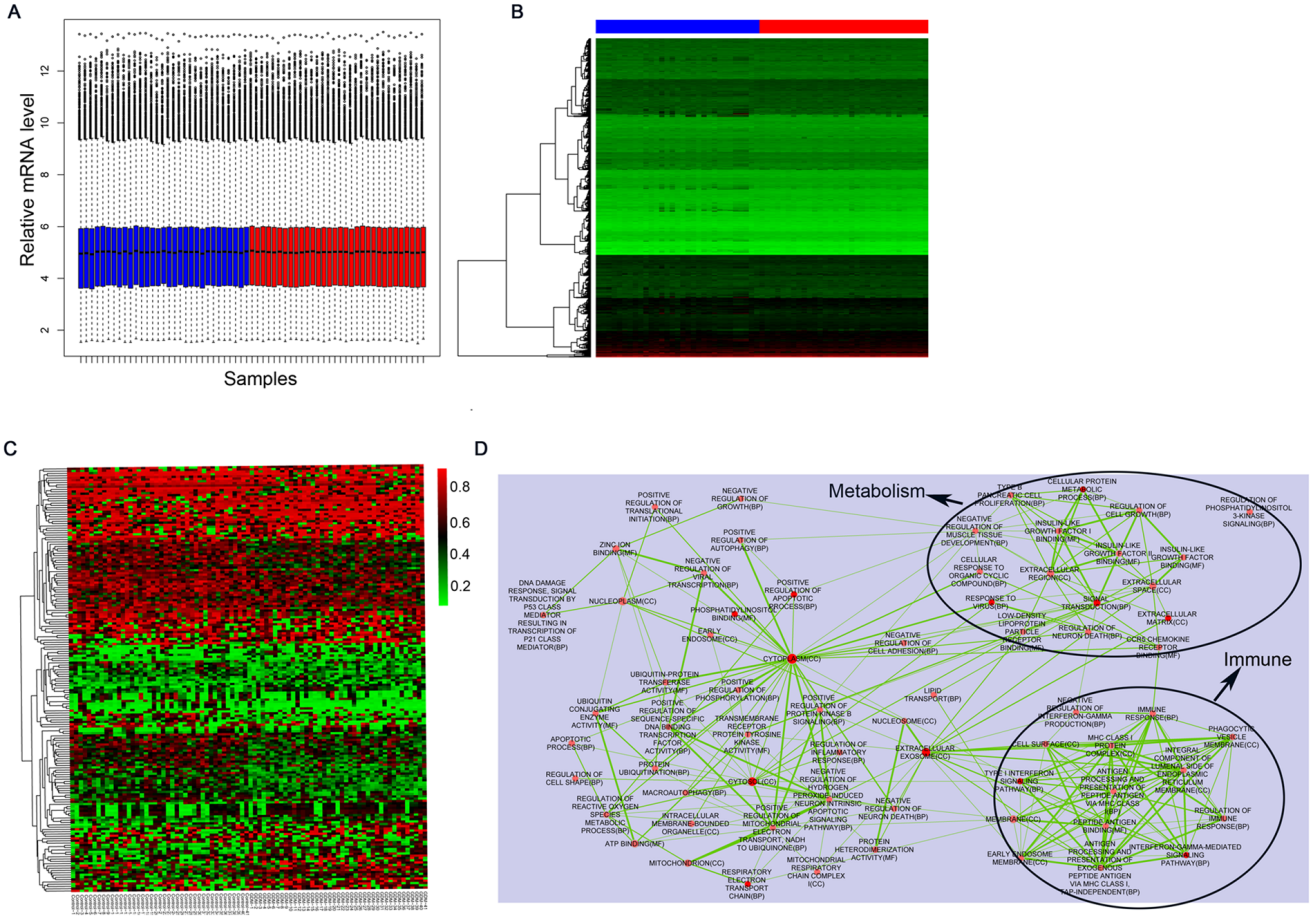
(**A**) Box plot depicts the overall expression level across samples after normalization. (blue bar: normal samples, red bar: GDM samples). (**B**) Heatmap shows the differential expressed genes’ expression profiles across samples under the standard of p < 0.05. Red in the scale bar represents high expression level, and green means relatively low expression level. c. Heatmap shows the 182 differential CpG methylation sites filtered by a more stringent criteria of p < 0.05 and |delta beta| > 0.2. Red in the scale bar represents sites with relatively higher methylation level and and green is the opposite. d. Cluster analysis of GO terms.

Raw data were normalized and overall gene expression level was comparable across samples (Fig. 2A), indicating the high quality of the original data. We performed differential expression analysis with all genes detected and identified 931 differential expressed genes (DEGs) between normal samples and GDM patient samples. Figure 2B illustrates the heatmap of DEGs’ expression profiles in normal and GDM patient samples with green and red color indicates low and high expression values respectively. What’s more, we screened out 182 DMSs satisfied |delta beta| > 0.2 from the total DMSs, and Fig. 2C is the heatmap of 182 DMSs’ beta value in normal and GDM patient samples.

There are 326 overlapping genes between DMG and DEG, and their expression are probably regulated by DNA methylation. To decipher the functional roles of these genes, we next conducted GO term enrichment analysis. GO Terms showed obvious enrichment on metabolism indicating strong links to diabetes as a metabolic disorder (Fig. 2D). In line with previous research^16^, our data also enriched terms associated with immune reactions indicating connections between insulin resistance and immune pathways. Growing evidence in the literature suggests that insulin resistance is the result of an inflammatory milieu^17^. IL-1 and TNF-α induce synergistic pleiotropic responses that profoundly affect the production of extracellular matrix (ECM) proteins. Increased expression of fibronectin, laminin β-1, and metalloproteinases may induce a fibrotic response and disrupt the structural integrity of placental endothelial cells as in other cell types^18^. We next queried the KEGG (Kyoto Encyclopedia of Genes and Genomes) databases with DMGs that were differentially expressed between normal tissues and patient tissues, the core KEGG pathways were also closely related to immune systems including Systemic lupus erythematous and Herpes simplex infection (Table 1).

**Table 1:** Significantly enriched KEGG pathways of overlaps between DEGs and DMGs.

Pathway names	Hits	P-value	Genes
Systemic lupus erythematosus	10	0.00194	HIST1H2AC, CD86, HIST1H2BD, HLA-DRB1, H2AFV, HIST1H2BG, HIST1H4F, HIST3H2A, C1R, HIST1H4D
Herpes simplex infection	11	0.00498	SP100, HLA-DRB1, TAP1, OAS1, HLA-C, NFKB1, CDC34, CCL5, HLA-E, STAT1, TBPL1
Transcriptional misregulation in cancer	10	0.00862	CD86, LMO2, FOXO1, BCL6, NFKB1, PBX3, MEIS1, ITGAM, WT1, CDK14
Ubiquitin mediated proteolysis	8	0.0248	RNF7, UBE2M, SIAH1, CDC34, UBE2QL1, UBE2Q2, BIRC3, UBE2B
Cell adhesion molecules (CAMs)	8	0.0294	CD86, HLA-DRB1, ITGB8, CLDN10, HLA-C, HLA-E, SELE, ITGAM
Graft-versus-host disease	4	0.0312	CD86, HLA-DRB1, HLA-C, HLA-E
Allograft rejection	4	0.0418	CD86, HLA-DRB1, HLA-C, HLA-E
Huntington’s disease	9	0.0485	SDHA, POLR2G, NDUFS6, NDUFB10, COX7A2, NDUFA10, TBPL1, SOD2, AP2M1

### Network Analysis

**Figure 3:**
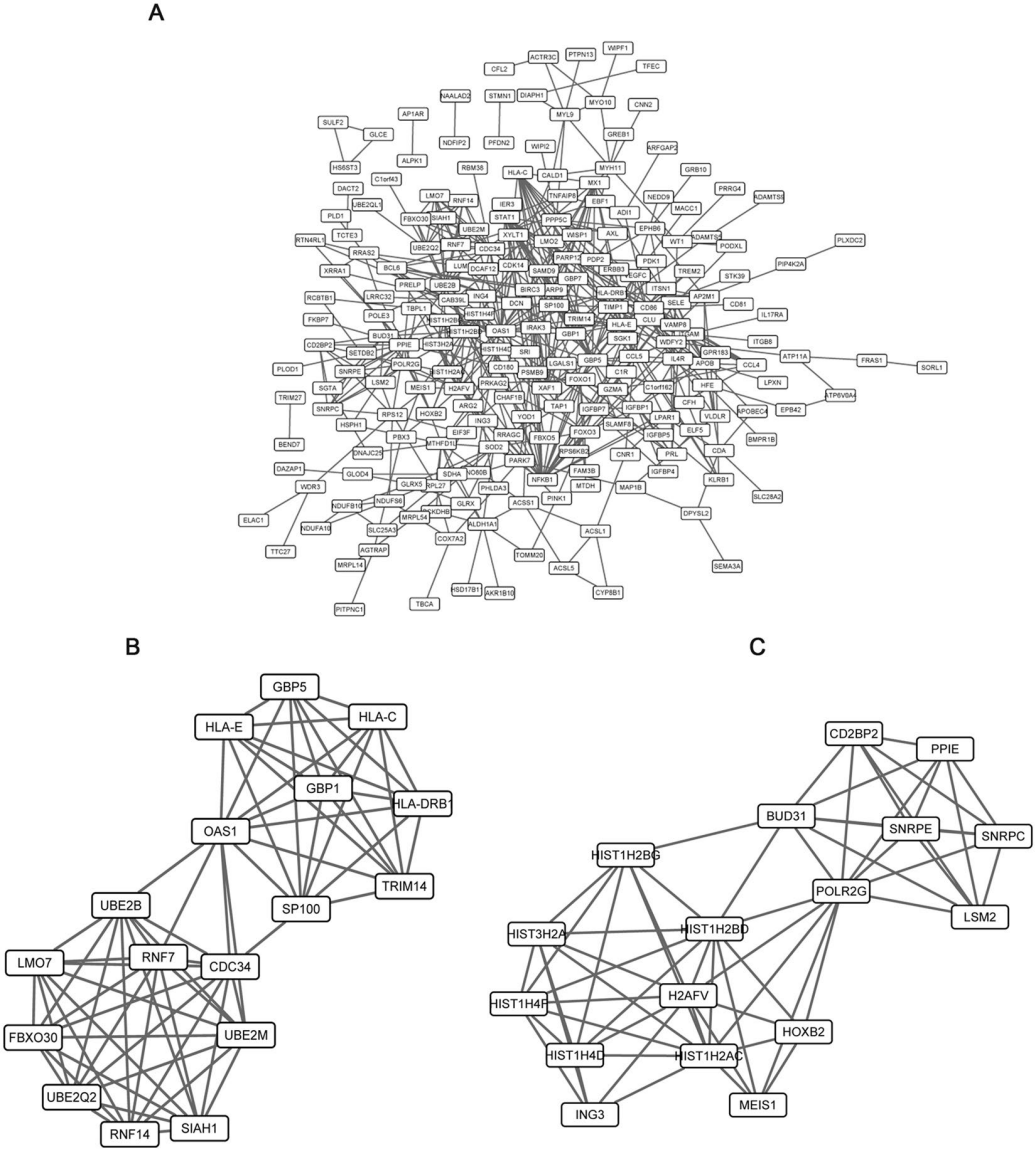
PPI network analysis of the overlaps between DEGs and DMGs. (**A**) overall network. (**B**,**C**) is the subnetwork module with module score >2.

**Figure 4:**
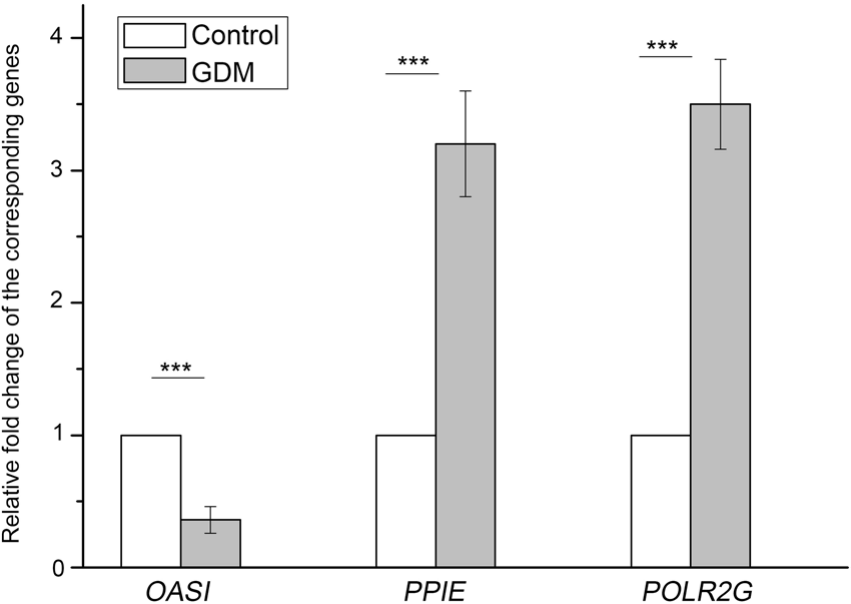
Relative mRNA level of *OAS1*, *PPIE* and *POLR2G* in placenta tissues of GDM patients and normal controls quantified by real time PCR.

To understand the interactive relationship of the overlapping genes between DMG and DEG, we performed network analyses using the STRING database. In general, genes with high STRING scores are often in a protein complex or parts of pathways. Besides, proteins involved in the same disease have an increased tendency to interact with each other^19^. It was reported that important target genes that related to the onset and development of a certain disease are always at the center of protein interaction network. To detect more efficient target genes, we performed modular analysis. As a result, we obtained a PPI network which contains 222 gene nodes and 539 edges (Fig. 3A), as well as 2 gene modules satisfied score >2 (Fig. 3B,C). *Oas1* has the highest node degree score in the PPI network and exists in our module (Fig. 3B). It was reported to be a downstream factor of IFN and involve in immune responses^20^, conforming with our pathway enrichment analysis. The other two factors, *Ppie* and *Polr2g* also get high scores and appear in the module (Fig. 3C). Pipe was accepted as a factor involves in metabolism^21^. Additionally, expression changes of *PIPE*, *POLR2G* and *OAS1* were further confirmed through real time PCR in placenta tissue of three GDM samples and three normal samples as shown in Fig. 4. Taken together, our research finds 3 GDM target genes and contributes new insights to the research and development of targeted drugs.

## Discussion

GDM patients are with higher risk to develop type 2 diabetes than pregnant women with normal blood glucose levels in the future^22^. Type 2 diabetes will be a major health burden without medical intervention properly. Besides, Pregnant women with gestational diabetes mellitus are prone to miscarriage during early pregnancy and the Impaired fetal development may lead to glucose intolerance and obesity of the baby^23^. Therefore, the early diagnosis of GDM and personalized medical intervention are of significant importance.

Studies on diabetes focus mostly on the pancreatic islets. The cell fate decision, maturation, the development of beta cell heterogeneity as well as aging and apoptosis were comprehensively studied. More and more omic data such as RNA-seq, DNA methylome, transcription factor and histone modification ChIP-seq are deciphering these cellular events. However, early stage prenatal diagnosis does not rely on the molecular released from islets of the pregnant women. So in-depth study of the molecular signatures on placenta is an urgent need when diagnosing GDM. Placenta is an important link between maternal and fetal nutrient exchange, and the mRNA of placental origin is readily detectable in maternal plasma^24^. Furthermore, the placenta are peripheral tissues that can be considered as a readout of the insulin resistance. Therefore, deciphering the transcriptome changes and corresponding epigenetic regulatory mechanism of planceta tissue in GDM patients is an ideal way to understand the pathology of the GDM and will shed light on GDM treatment and T2D prevention.

Our work deeply interprets the DNA methylome and gene expression data with normal placenta tissue and GDM patient placenta tissue, and find a list of genes that may be regulated by the epigenetic way. The development of dcas9 (deficient CRISPR-associated 9 nuclease) technique provides the possibility for the site-specific edition of epigenetic modifications^25–27^. Our research has dug out three genes, namely *Oas1, Ppie, Polr2g*. Further studies will explore the regulatory strategies for the expression level of these genes and try to reveal the roles of these proteins in their interaction network. We can edit important target genes by dcas9 or other epigenome editing method to alleviate the harmful effect brought to fetal development by GDM. Moreover, women with GDM can take specific small-molecule inhibitors targeting pathogenic genes during pregnancy to alleviate symptoms and reduce the chances for their children to get metabolic disorders in the future.

In conclusions, our study identified several biological processes and genes that might contribute the development of GDM, this should be helpful for its diagnosis and targeted therapy.

## Materials and Methods

### Microarray datasets

The microarray datasets in this study were downloaded from the Gene Expression Omnibus (GEO, http://www.ncbi.nlm.nih.gov/geo/). The gene expression (GSE70493) and DNA methylation profiles (GSE70453) were deposited by Binder *et al*. and composed with the same samples: 32 GDM patient placenta tissue and 31 normal samples^28^. The genome-wide expression profiles were quantified by the commercial gene microarray GeneChip® Human Transcriptome Array 2.0 (Affymetrix). Methylation profiling data was obtained through the Illumina Infinium HumanMethylation450 Beadchips.

### Microarray Analysis

Methylation data was preprocessed for the reliability of the following analyzes, which mainly involve the filtering of methylation sites with detection P-value > 0.05 in more than 20% samples and samples contains more than 75% methylation sites with detection P-value > 0.05. Methylation level of CpG site is represented by beta value, which calculated as (methylated signal intensity)/(100 + methylated signal intensity + unmethylated signal intensity). Methylation level for specific region, such as the promoter of a gene, is the average beta value of all CpG sites contained in this region. Differential methylation analysis at site-level and region-level analysis were performed using IMA package with the criteria of P-value < 0.05 for the identification of differential methylation sites (DMS) and regions (DMRs).

We then conducted differential expression analysis between normal person and patients with Gestational diabetes Mellitus. Briefly, the raw data were imported into R and normalized via the oligo package. Then the limma package was used for the screening of differential expression genes (DEGs) with the criteria of P-value < 0.05.

### Functional clustering analysis

We conducted the Gene Ontology (GO) and Kyoto Encyclopedia of Genes and Genomes (KEGG) pathway enrichment analysis of DEGs and differential methylation genes (DMGs, i.e. genes contain at least one DMS) based on the Database for Annotation, Visualization and Integrated Discovery (DAVID, http://david.abcc.ncifcrf.gov/). The thresholds used in this study is P-value < 0.05.

### Protein-protein interaction network analysis

We performed protein-protein interaction (PPI) network analysis for the overlaps between DEGs and DMGs based on the STRING database (STRING, https://string-db.org/) with the criteria of comprehensive score >0.4. Protein-protein interaction was visualized with Cytoscape (http://cytoscape.org/). For the interpretation of PPI network, we conducted modular analysis with MCODE plugin, which could identify protein clusters that might represent protein families and parts of pathways through seeking dense regions of network, of Cytoscape and identified network modules with score >2.

### Real time PCR

Real time PCR was performed for validating expressions of *OAS1, PPIE* and *POLR2G*. Placenta tissues from three GDM patients and three normal samples were collected and subjected to RNA extraction. The concentration and purity of the RNA were determined by spectrophotometer (Spectrophotometry GE). The total RNA obtained was stored in a −80 °C refrigerator, and then reverse-transcribed into cDNA using the PrimeScript RT kit according to the instructions. The obtained cDNA could be used as a template to detect the amount of mRNA of the corresponding gene. Primers of those three genes were as follows: *POLR2G* forward: GACCTGCACAGGGAAGTATGGC, reverse: CTGGATAAAGGACAAAGCCTCGG; *OAS1* forward: GGATTCTGCTGGCTGAAAGCAAC, reverse: GGAGTGTGCTGGGTCTATGAGAG; *PPIE* forward: GGGCAAGTCCATCTATGGGAAG, reverse: GGATAGTAGACCTGGTCCCGTA. This study was approved by the Ethics Committee of the Shanghai family planning hospital and written informed consent was obtained from all participants.

## Data Availability

The datasets generated and analyzed during the current study are available from the corresponding author on reasonable request.
